# Robot-assisted gait training for improved gait independence in individuals with acute hemiparetic stroke: study protocol for a randomized controlled pilot trial

**DOI:** 10.1186/s40814-025-01694-6

**Published:** 2025-08-29

**Authors:** Daisuke Kato, Satoshi Hirano, Daisuke Imoto, Takuma Ii, Daisuke Matsuura, Takuma Ishihara, Yohei Otaka

**Affiliations:** 1 Department of Rehabilitation, Fujita Health University Hospital, 1-98 Dengakugakubo, Kutsukake-Cho, Toyoake, Aichi 470-1192 Japan; 2 Department of Rehabilitation Medicine, School of Medicine, Fujita Health University, 1-98 Dengakugakubo, Kutsukake-Cho, Toyoake, Aichi 470-1192 Japan; 3 Faculty of Rehabilitation, School of Health Sciences, Fujita Health University, 1-98 Dengakugakubo, Kutsukake-Cho, Toyoake, Aichi 470-1192 Japan; 4 Innovative and Clinical Research Promotion Center, Gifu University Hospital, 1-1 Yanagido, Gifu, 501-1194 Japan

**Keywords:** Cerebrovascular disease, Gait ability, Rehabilitation, Robotics, Stroke, Stroke rehabilitation, Walking, Feasibility studies, Randomized controlled trials as topic

## Abstract

**Background:**

Robot-assisted gait training (RAGT) has proven effective in addressing gait disorders in patients with stroke. However, its efficacy in patients with acute stroke has not yet been demonstrated. This pilot study is designed to evaluate the following: (1) feasibility of conducting a randomized controlled trial on RAGT for enhancing gait postacute stroke and (2) to obtain preliminary estimates regarding the potential efficacy of RAGT for achieving gait independence during the acute phase.

**Methods:**

We will conduct an assessor-blinded, single-center, randomized controlled pilot trial involving 32 patients with acute stroke who are unable to walk. Participants will be randomly assigned to either the RAGT or the conventional gait training (CGT) groups. Each participant will receive 180 min of daily rehabilitation, including 60 min dedicated to gait training. The RAGT group will receive 40 min of RAGT and 20 min of CGT, while the CGT group will engage in 60 min of CGT. Interventions will continue for up to 8 weeks, or until participants achieve gait independence, as indicated by a Functional Ambulation Category score of ≥ 3. Feasibility outcomes will include recruitment, enrollment, protocol adherence, and retention rates. The primary clinical outcome will be the incidence of achieving gait independence during the intervention period. Secondary clinical outcomes will include gait performance measures, assessments of physical function and activity, and intervention dose. Adverse events associated with RAGT and CGT will also be documented to evaluate the safety of both interventions.

**Discussion:**

Implementing RAGT during the acute phase of stroke may facilitate earlier attainment of gait independence compared to CGT. We aim to provide valuable insights into the feasibility of the proposed study design and generate preliminary data on the potential effects of RAGT on gait independence in the acute phase of stroke, providing a framework for future larger-scale trials.

**Trial registration:**

This clinical trial was registered with the Japan Clinical Trials Registry (jRCT) on 19 June 2023 (registration number: jRCTs042230040). The study protocol was initially registered as version 1.0 and has since undergone minor amendments—currently on version 4.0. This protocol was written based on the latest version (ver. 4.0) registered with jRCT.

## Background

Stroke is a major cause of physical dysfunction and gait disorders [1, 2]. Gait disorders limit activities of daily living and social participation [3]. The percentage of individuals recovering their gait ability post-stroke is estimated to be 41–85% [4]. Therefore, enhancing gait function in stroke survivors is an important goal of rehabilitation therapy [5].

Recent literature has increasingly reported the efficacy of robot-assisted gait training (RAGT) in individuals with stroke. A systematic review and meta-analysis indicated that RAGT, when combined with conventional physical therapy (CGT), significantly improved gait independence in individuals who were unable to walk within 3 months of stroke onset [6], leading to its inclusion in several guidelines [7, 8]. The efficacy of RAGT for individuals with hemiparetic stroke is attributed to the robotic control mechanism, which facilitates intensive repetitive training and task-oriented training by providing partial or full body weight support and assistance with movement [9].

However, the optimal timing for initiating RAGT remains unclear. Most studies evaluating the efficacy of RAGT have focused on individuals with stroke in the subacute phase. The aforementioned systematic review and meta-analysis concentrated on randomized controlled trials involving participants 2–8 weeks following stroke onset [6]. Other systematic reviews have consistently demonstrated the efficacy of RAGT in enhancing gait ability in individuals within the first 3 months post-stroke, yet they did not include randomized controlled trials conducted during the first week post-stroke [10–12]. Further research is required to determine the appropriate timing for implementing RAGT [13].

We defined the “acute” phase as the period occurring within 7 days of stroke onset [14] and have planned a study to clarify the efficacy of RAGT initiated during this timeframe. We hypothesize that the initiation of RAGT in the acute phase would enable individuals to achieve gait independence more rapidly than with CGT. Early RAGT promotes motor learning and mitigates disuse by delivering high-intensity, task-oriented gait training from an early stage [9, 15]. To test this hypothesis, we aim to examine the following: (i) the feasibility of conducting a randomized controlled trial on RAGT for improving gait after acute stroke and (ii) obtaining preliminary estimates of the potential efficacy of RAGT for achieving gait independence during the acute phase.

## Methods

### Study design and setting

**Fig. 1:**
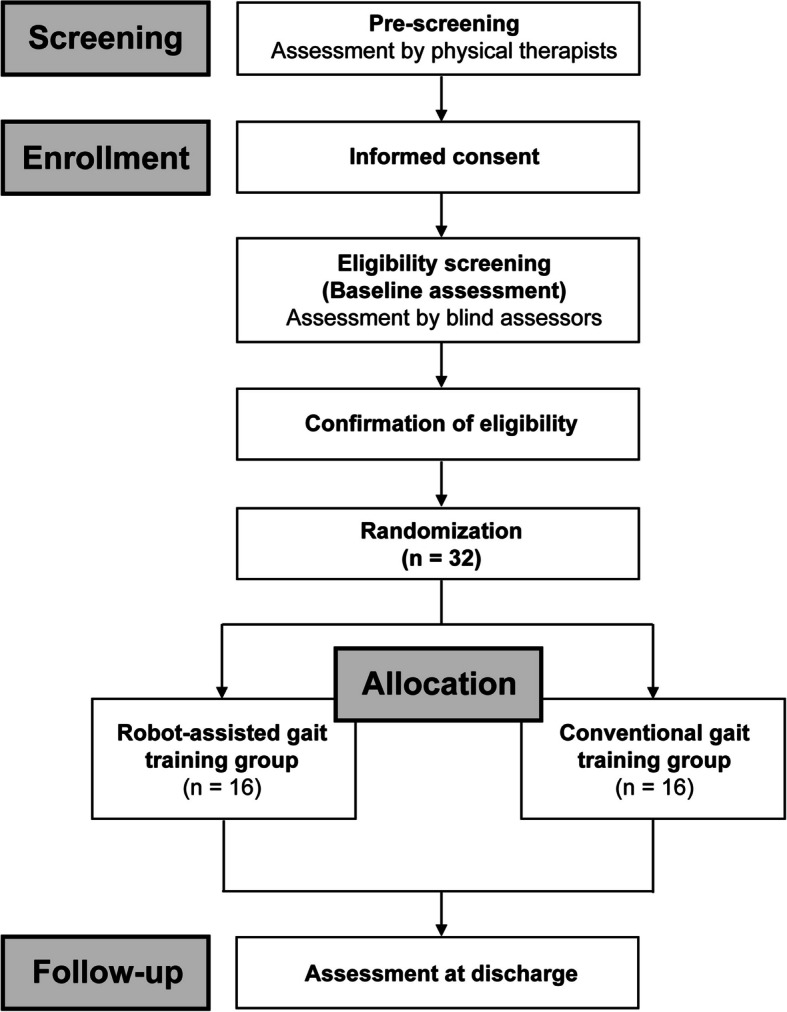
Study flowchart

This study is an assessor-blinded, single-center, randomized controlled pilot trial following the Consolidated Standards of Reporting Trials (CONSORT) extension for pilot trials [16]. A flowchart of the study is illustrated in Fig. 1. Individuals with stroke will be enrolled and randomly assigned to either the RAGT group or the CGT group. The study will be conducted at Fujita Health University Hospital, Aichi, Japan. In accordance with the Declaration of Helsinki of 1964 (revised in 2013), written informed consent will be obtained from all participants (or their kin or legal guardians if the participants are unable to provide consent) prior to participation. This study received approval from the Fujita Health University Institutional Review Board (Approval No. CR24-031) and is registered in the Japan Registry of Clinical Trials (jRCTs042230040).

### Participants

The inclusion criteria are as follows: (i) individuals aged 16–90 years, (ii) those with hemiplegia due to a first-ever supratentorial cerebral infarction or hemorrhage, (iii) those starting intervention within 7 days of stroke onset, (iv) those with severe lower extremity hemiparesis at the preintervention assessment (total score of ≤ 6 on the Stroke Impairment Assessment Set [SIAS] [17–19] motor items for lower extremities), (v) those with severe gait disorder at the preintervention assessment (score of ≤ 1 on the Functional Ambulation Categories [FAC] [20, 21]), (vi) those with sustained awareness at the preintervention assessment (eye opening score of 4 and motor response score of 6 on the Glasgow Coma Scale [GCS] [22, 23]), (vii) those with body weight of 35–100 kg, and (viii) those with height of 140–190 cm.

The exclusion criteria are as follows: (i) individuals with a history of stroke (except for individuals with no symptoms despite imaging findings indicating a history of stroke); (ii) those with pre-existing gait disorders due to neuromuscular or musculoskeletal diseases; (iii) those that participated in other interventional studies related to lower limb/trunk motor function and gait; (iv) those with a history of epileptic seizures within the past 2 years (including seizures caused by the current stroke); (v) those with cardiovascular or respiratory diseases that impede exercise therapy; (vi) those with uncontrolled hypertension; (vii) those with osteoporosis of the lower limbs or spine; (viii) those with heterotopic ossification in the lower extremities leading to limited range of joint motion; (ix) pregnant women or those with possibility of pregnancy; (x) those with infectious diseases requiring isolation; (xi) those with urinary or fecal incontinence that may compromise the robotic device through contamination; (xii) those unable to wear the robotic leg due to overweight, deformities in lower limbs, or pressure ulcers; and (xiii) individuals deemed unsuitable by the attending physician, principal investigator, or research assistants.

### Recruitment

Potential participants will be identified from patients admitted to the Fujita Health University Hospital Emergency Center. Prescreening of potential participants will be conducted by trained physical and occupational therapists, and eligibility will be carefully assessed by a rehabilitation physician based on the prescreening results.

### Randomization

Participants will be stratified by stroke type and randomly allocated to either the RAGT or CGT group using a computer-generated permuted block randomization method with permuted block sizes of two and four. Random allocation will be performed by an independent researcher not involved in the assessments, using the Research Electronic Data Capture tool [24].

### Interventions

**Fig. 2:**
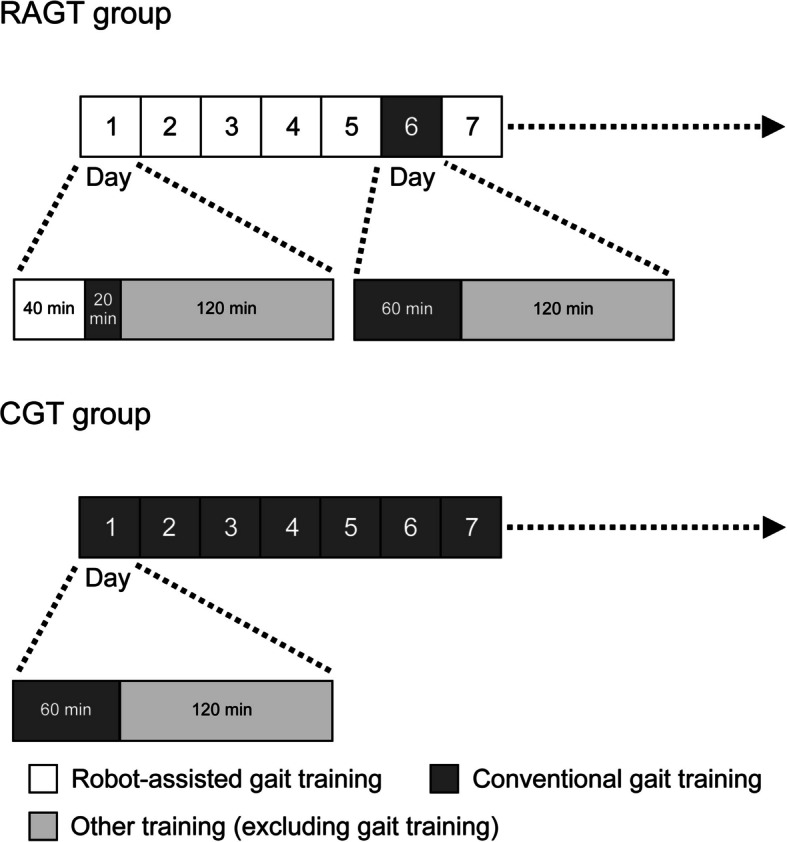
Intervention protocol. The intervention will be conducted 7 days a week until the Functional Ambulation Category score is ≥ 3 or until 8 weeks from the start of training. The participants will receive 180 min of rehabilitation per day. The daily gait training for both groups will last 60 min, and the remaining 120 min of rehabilitation will comprise other training (excluding gait training). In the robot-assisted gait training (RAGT) group, 40 min of RAGT and 20 min of conventional gait training (CGT) will be provided 6 days a week (days of RAGT and CGT), and 60 min of CGT will be provided once a week (in principle, only CGT will be provided on Sundays). In the CGT group, patients will receive 60 min of CGT for 7 days a week

The study schedule is illustrated in Fig. 2. Participants will undergo the intervention during their hospitalization at Fujita Health University Hospital. The intervention will continue until participants achieve an FAC score of ≥ 3 or for up to 8 weeks from the start of training. Participants in both the RAGT and CGT groups will receive rehabilitation for 180 min per day, 7 days per week. Both groups will undergo gait training for 60 min per day [30] and non-gait training for 120 min per day. The RAGT group will undergo RAGT for 40 min per day and CGT for 20 min per day, for 6 days a week, with an additional 60 min of CGT once per week. The CGT group will undergo CGT for 60 min, 7 days per week (Fig. 2).

The following medical devices and therapies are prohibited during the intervention period: medical devices, such as electrical, magnetic, and shock wave stimulators for the lower limbs; other devices for improving gait and balance; vibration stimulators; virtual reality devices; augmented reality devices; mixed reality devices; therapies such as mirror therapy or constraint-induced movement therapy for the lower limbs.

### Robotic device

**Fig. 3:**
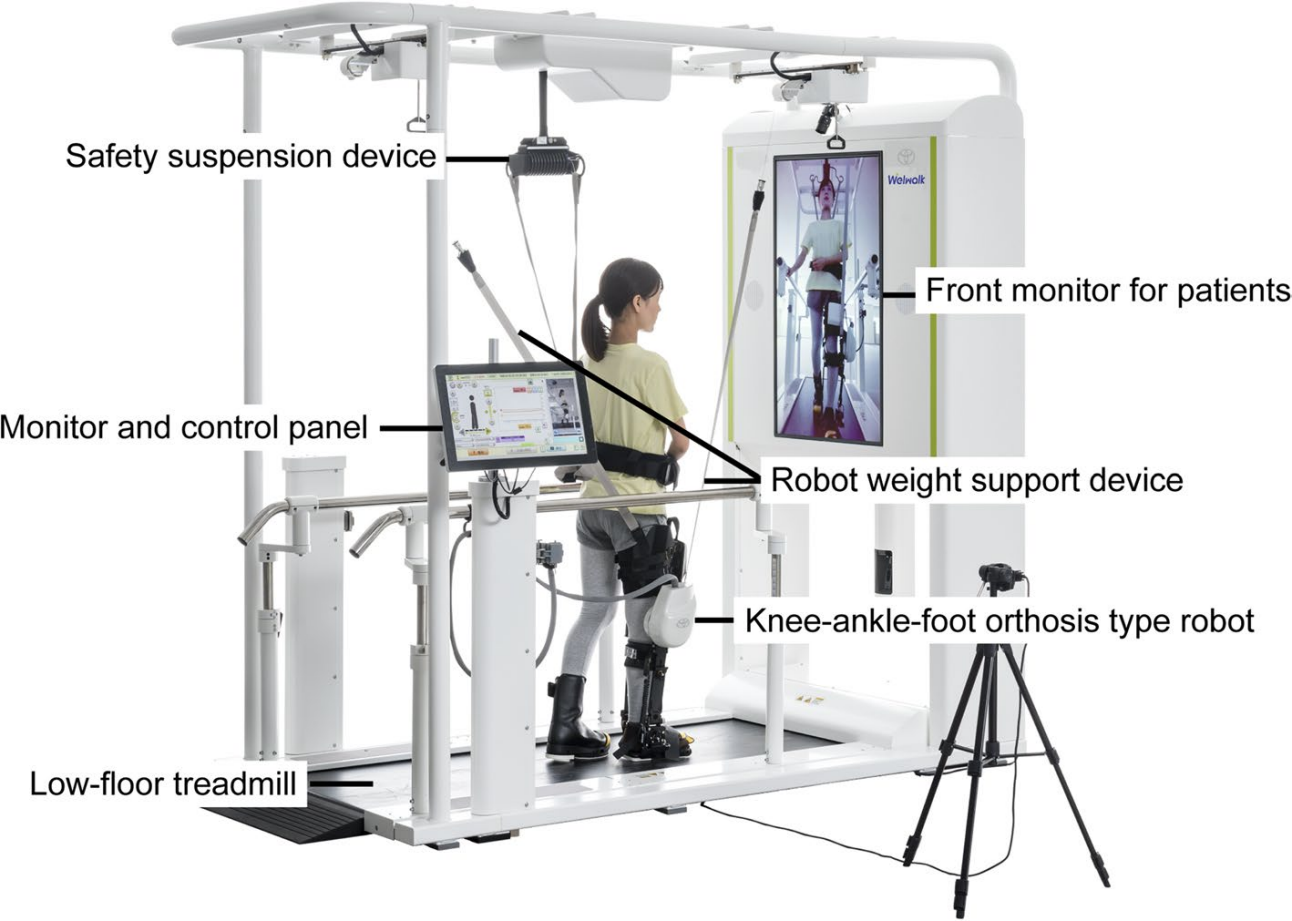
Overview of the Welwalk device

The Welwalk (Toyota Motor Corporation, Aichi, Japan), which was developed to support gait training in individuals with hemiparetic stroke, will be used for RAGT (Fig. 3). A multi-center randomized controlled study has reported that RAGT, using Welwalk, is effective in improving gait independence in individuals with subacute cerebral infarction [25]. Several single-center randomized controlled studies have similarly reported improvements in gait independence through RAGT with Welwalk in individuals with subacute stroke [26, 27]. The device consists of a low-floor treadmill, knee-ankle-foot orthosis-type robot, safety suspension system (used for body weight support), robot weight support device, front monitor for patients, and monitor and control panel for the therapist. The knee-ankle-foot orthosis-type robot is equipped with a load sensor on the sole that detects the gait cycle based on the load. The knee joint motor assists in knee joint flexion during the swing phase and extension during the stance phase. Therapists will set the degree of robotic assistance as low as possible to avoid complicating gait mechanics significantly while still allowing for compensatory movements. Furthermore, therapists will provide minimal support or guidance as needed.

### Outcomes

#### Feasibility assessment

Following prior recommendations [28], we will assess the feasibility of this pilot study based on the following indicators: (i) recruitment and enrollment rates, defined as the average number of participants enrolled in the study per month; (ii) enrollment rate, defined as the proportion of individuals who consent to participate among those who meet the eligibility criteria during the preintervention assessment; (iii) adherence rate, defined as the proportion of participants who complete the intervention according to the schedule, serving as an indicator of the intervention’s acceptability; and (iv) retention rate, defined as the proportion of participants who complete outcome measures from baseline to predischarge assessment.

These measures will serve as progression criteria to determine whether to proceed with the study [29]. The specific criteria are as follows: (i) recruitment rate of at least 0.8 participants per month; (ii) enrollment rate of at least 90% of screened eligible individuals consenting to participate [30]; (iii) adherence rate, with at least 90% of participants completing scheduled training times [30]; and (iv) retention rate, with a minimum of 90% of enrolled participants completing all outcome measures until follow-up [30, 31]. Recruitment rate criteria were established based on our institution’s experience, while enrollment, adherence, and retention rates were determined with reference to similar previous studies on RAGT [30, 31]. Even if a participant fails to meet any of the four established criteria, the study will proceed with protocol modifications if the underlying issues can be addressed [29].

#### Safety assessment

To evaluate the safety of the intervention, the occurrences of serious adverse events and treatment-related adverse events associated with RAGT and CGT will be recorded. Serious adverse events will be monitored for five specific events based on the guidelines [32]. Upon confirmation of a serious adverse event, the principal investigator will promptly report it to the Institutional Review Board and the Ministry of Health, Labour and Welfare. Treatment-related adverse events will be documented for occurrences such as skin injuries and falls. In the event of an adverse event, the therapist responsible will prepare an adverse event report that will include the name of the adverse event, the date of incidence, a detailed description of the course of the adverse event, the treatment for the adverse event, and the outcome following treatment. A report will be generated for each serious adverse event or treatment-related adverse event and compiled upon participant discharge.

#### Clinical outcomes

**Fig. 4:**
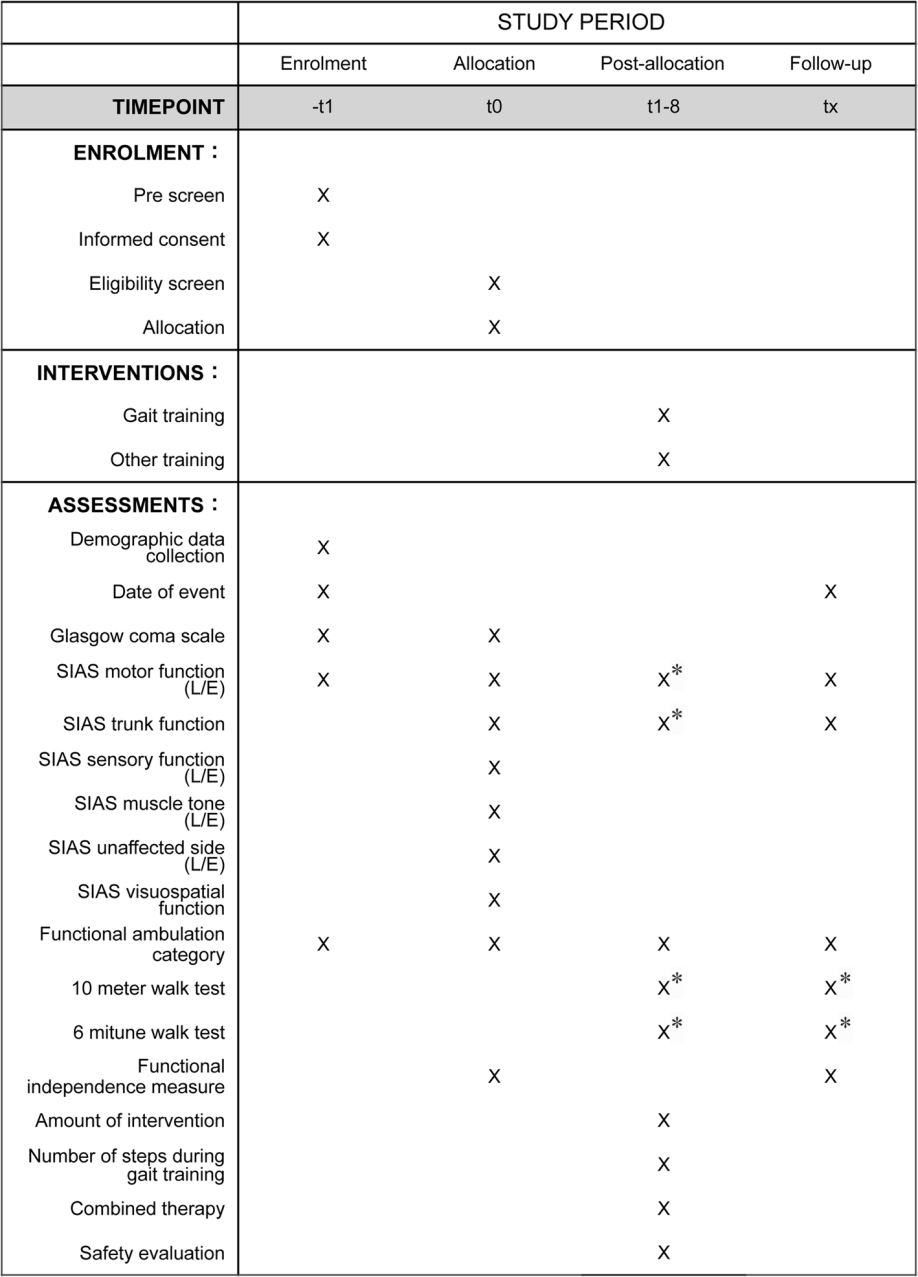
SPIRIT figure Note: * Items to be assessed if the Functional Ambulation Category score is ≥3. SIAS, Stroke impairment assessment set

The Standard Protocol Items: Recommendations for Interventional Trials (SPIRIT) diagram presented in Fig. 4 summarizes the data collection schedule. Furthermore, we have included the SPIRIT 2013 checklist as an additional file, comprising recommended items to address in a clinical trial protocol. Blinded assessors will perform assessments weekly after the baseline assessment until participants achieve an FAC score of ≥ 3. Continuous weekly assessments will be terminated once the participants achieve an FAC score of ≥ 3 or when they undergo the intervention for 8 weeks. However, assessments at 4 and 8 weeks and discharge will always be performed, regardless of whether an FAC score of ≥ 3 is achieved. The blinded assessments will be conducted by three physical therapists with > 10 years of experience. The assessors will confirm the assessment methods sufficiently prior to the commencement of the study.

The primary clinical outcome measures will include the number of days from the baseline assessment to the achievement of gait independence and the percentage of individuals achieving gait independence—operationally defined as an FAC score ≥ 3. In particular, this primary clinical outcome represents the most promising candidate for future clinical trial endpoints. Secondary clinical outcomes encompass gait parameters as assessed by velocity, stride length, and cadence in the 10-m walk test; 6-min walk distance; SIAS scores for lower limb motor and trunk function; total score on the Functional Independence Measure (FIM); and the number of steps taken during gait training during the intervention period. The FAC is a reliable and validated clinical measure of gait ability based on the amount of assistance needed [20, 21]. The 10-m walk test is a reliable and validated clinical measure of walking ability [33, 34], whereas the 6-min walk test is a reliable and validated clinical measure of motor endurance [35, 36]. The number of steps taken during gait training will be recorded manually by a therapist using a counting device. The SIAS serves as a reliable and validated index for assessing functional impairment in patients with stroke [17–19]. It accounts for motor function, tension, sensory function, range of motion, pain, trunk function, higher brain function, and muscle strength on the non-paralyzed side, with total scores ranging 0–72. The total score for SIAS motor items is calculated as the sum of the hip flexion, knee extension, and foot pat tests, with scores ranging 0–15 points. The FIM, which comprises 13 motor items and 5 cognitive items, is a proven reliable index for assessing independence in activities of daily living [37, 38]. Each item is rated on a scale of 1–7, with scores of 1–4 indicating the need for varying levels of physical assistance, a score of 5 indicating the need for supervision, a score of 6 indicating modified independence, and a score of 7 indicating complete independence. The FIM cognitive total is the sum of scores of the five cognitive items, ranging 5–35 points.

In addition, to understand participant characteristics, the following data will be collected at baseline: age; sex; height; weight; disease type; affected side; days post-stroke onset at admission, assessment, and discharge; National Institutes of Health Stroke Scale (NIHSS) score; GCS score; SIAS motor function scores for the lower extremities; trunk function; sensory function in the lower extremities; tone in the lower extremities; function in the lower extremities on the unaffected side; and visuospatial function. The NIHSS is a reliable and validated index for assessing stroke severity [39–41]; it evaluates consciousness, gaze, visual field, facial paralysis, paralysis of the extremities, ataxia, sensory disturbance, aphasia, dysarthria, elimination issues, and neglect, with scores ranging 0–42. A score of 0 is considered normal, while 42 indicates the most severe condition. The GCS consists of three items, namely eye opening, verbal response, and motor response. Eye opening is scored from 1 to 4, verbal response from 1 to 5, and motor response from 1 to 6. The total GCS score is defined as the sum of the scores of these three items, ranging 3–15, with 3 indicating severe impairment and 15 indicating normal responsiveness.

### Data management

Research data will be entered and managed using REDCap (Research Electronic Data Capture) hosted by Fujita Health University. Identifiable personal information will be deleted, and only coded and anonymized data will be used. Data will be password-protected, and access will be restricted to authorized research personnel. Data quality will be improved through predefined range checks and routine monitoring by the research team.

### Sample size

This is a pilot study; therefore, the sample size was not calculated based on statistical testing power [16]. We aim to enroll 32 participants, as this is presumed to be an adequate sample size for gathering insights on recruitment, enrollment, adherence, and retention rates. With 32 participants recruited, a retention of 90% can be estimated to within a 95% confidence interval (CI) ranging from 75–96%. This sample size should also suffice to accurately assess means and variances [42] and to provide data regarding clinical outcomes.

### Blinding

Due to the differing treatment programs between the RAGT and CGT groups, it will be challenging to blind participants and therapists with respect to group assignment. Therefore, only the assessors will remain blinded. To quantify the effectiveness of blinding, we will ask the blinded assessors to identify each participant’s group assignment at the conclusion of all assessments.

### Analytical methods

Feasibility results will be reported using descriptive statistics. Recruitment, enrollment, adherence, and retention rates will be computed with 95% confidence intervals. The adverse events and their frequencies will be recorded. Any exclusions or dropouts, along with their reasons and stages of occurrence, will also be recorded. The success of blinding will be detailed as a percentage of correct guesses.

The primary outcome measure will be the incidence of gait independence during the intervention period. The cumulative incidence of gait independence will be analyzed using the Kaplan–Meier method, and the log-rank test will evaluate the significance of differences in the Kaplan–Meier curves between groups. Cumulative incidence of gait independence events at the end of the intervention, along with the median duration from baseline to gait independence attainment, and the restricted mean survival time until the end of the intervention will be calculated. Additionally, to supplement the initial treatment effect analysis, differences will be evaluated using the generalized Wilcoxon test. Individuals who do not achieve gait independence during the intervention period will be censored. Secondary clinical outcomes will encompass gait performance measures, physical function, and activity outcomes at baseline, 4 weeks, 8 weeks, and discharge, alongside the intervention dose. Comparisons between the two groups will utilize the chi-square test for categorical variables and either Student’s *t-*test or Mann–Whitney *U* tests for continuous variables. To evaluate the potential effectiveness of the intervention and gather data useful for future trial design, we will estimate the effect size for the time elapsed from baseline to achieving gait independence, specifically estimating the hazard ratio as a relative measure of the likelihood of achieving gait independence over time.

## Discussion

A systematic review and meta-analysis demonstrated the effectiveness of early repetitive gait training in enhancing gait independence in patients with stroke [43]. RAGT facilitates intensive, repetitive, and task-oriented training for individuals with hemiparetic stroke by partially or completely supporting their weight and movement through a robotic control mechanism [9]. Therefore, various reports have indicated that RAGT also facilitates repetitive gait training for individuals with stroke who are unable to walk [6, 7].

The objectives of this pilot study are as follows: (i) to evaluate the feasibility of performing a randomized controlled trial on RAGT for improving gait ability following acute stroke and (ii) to obtain preliminary estimates of the potential efficacy of RAGT for improving gait independence during the acute phase. These findings will inform decisions related to the feasibility of future trials and, if necessary, guide protocol revisions. Potential adjustments could encompass modifying eligibility criteria to improve recruitment and enrollment rates, refining intervention parameters to boost adherence, or revising assessment schedules to address retention rates.

There are certain limitations of this trial protocol. First, the trial protocol is based on a small sample size, which may not provide sufficient statistical power to evaluate the effectiveness of the intervention conclusively. Therefore, the findings related to clinical outcomes should be interpreted as exploratory in nature. To address this, future studies should include a larger sample size to increase statistical power and the reliability of results, potentially through multi-center trials. Rather than directly using effect sizes obtained from this study to determine the sample size for a future definitive trial, we intend to use estimates of variability and feasibility indicators—including recruitment, adherence, and retention rates—as well as the minimally important difference, if identifiable, to inform future trial designs [44]. Second, the absence of long-term follow-up assessments in this pilot study limits the ability to evaluate the sustainability of intervention effects, including their impact on long-term functional independence and overall health. Future definitive trials should incorporate follow-up assessments beyond discharge to assess the durability and long-term outcomes of RAGT.

To the best of our knowledge, this will be the first study to investigate the effects of RAGT on gait independence improvement in individuals with acute stroke. We posit that RAGT will enhance gait independence in this population, and the comprehensive results of this investigation and future large-scale trials will constitute essential evidence regarding the potential benefits of RAGT as an intervention for individuals with acute stroke.

## Trial status

### Protocol information

- Protocol version number: 4.0
- Protocol version date: August 22, 2024.

### Recruitment timeline

- Recruitment start date: December 1, 2023.
- Expected recruitment completion date: March 31, 2026.

## Data Availability

The dataset is accessible only to authorized research personnel; however, the data used and analyzed in this study can be provided upon reasonable request to the corresponding author.

## Abbreviations

CGT: Conventional gait training
FAC: Functional Ambulation Category
FIM: Functional Independence Measure
GCS: Glasgow Coma Scale
NIHSS: National Institutes of Health Stroke Scale
RAGT: Robot-assisted gait training
SIAS: Stroke Impairment Assessment Set
